# Adjunctive Use of PerioCream Following Scaling and Root Planing: Preliminary Findings From an Observational Clinical Study

**DOI:** 10.7759/cureus.92160

**Published:** 2025-09-12

**Authors:** Andrea Mascolo, Silvia Cotellessa, Isabella Di Tanna, Oana Dinculescu, Elisabetta Ferrara, Ben Mettepenningen, Jessica Bassignani

**Affiliations:** 1 Academics, European Institute for Medical Studies (EIMS), St. Julian’s, MLT; 2 Dental Hygiene, Private Practice, Lanciano, ITA; 3 Dental Hygiene, Private Practice, Pescara, ITA; 4 EIMS Research Hub, European Institute for Medical Studies (EIMS), St. Julian’s, MLT; 5 Dental Hygine, Telematic University Leonardo da Vinci (UNIDAV), Torrevecchia Teatina, ITA; 6 Research and Development, Bonyf AG, Vaduz, LIE

**Keywords:** bleeding index, hrql, inflammation modulation, nitradine, oral hygiene adjunct, patient-reported outcomes, periocream, periodontal therapy, real-world evidence, vas

## Abstract

Background

Scaling and root planing (SRP) remains the standard of care for non-surgical periodontal therapy, but its long-term effectiveness is often limited by microbial recolonization and persistent inflammation. These challenges have led to growing interest in adjunctive topical agents that can enhance healing, reduce discomfort, and improve patient adherence. PerioCream, a mucoadhesive formulation based on NitrAdine®, has shown broad-spectrum antimicrobial activity, anti-inflammatory effects, and modulation of the oral microbiota in both laboratory and early clinical studies.

Objective

This study aimed to evaluate the adjunctive use of PerioCream after SRP in a real-world clinical setting, focusing on clinical outcomes, patient-reported experiences, and artificial intelligence (AI)-based assessment of tissue healing.

Methods

Fifteen adult patients (8 females, 7 males; aged 35-64 years) with moderate-to-severe periodontitis (Stage II/III, Grade B/C) underwent SRP followed by adjunctive topical application of PerioCream. Clinical parameters, including Bleeding on Probing (BOP) and Gingival Index (GI), were assessed at baseline (T0) and after 72 hours (T3). Patient-reported outcomes were recorded using a Visual Analog Scale (VAS) for discomfort and a Health-Related Quality of Life (HRQOL) questionnaire. Standardized intraoral images were analyzed using a convolutional neural network (CNN)-based AI model to evaluate visible signs of inflammation.

Results

All 15 patients completed follow-up without adverse effects. Clinical indices improved significantly: BOP decreased from 72% (70.5-74.0) to 12% (11.0-13.5) and GI from 1.6 (1.55-1.7) to 0.5 (0.4-0.5) (Wilcoxon W = 0, Z = −3.41, p < 0.001 for both). The VAS pain showed a numerical reduction from 3.53 to 2.27 (Wilcoxon W = 21.0, Z = 1.42, p = 0.157). AI-assisted image analysis indicated decreased inflammation in 13/15 patients, consistent with clinical findings.

Conclusion

The adjunctive use of PerioCream after SRP may be associated with early improvements in clinical outcomes and patient comfort; however, these findings are preliminary and require confirmation in larger controlled trials. The combination of traditional measures with AI-assisted image analysis offers a multidimensional perspective on healing and may support more personalized periodontal care. These findings provide a rationale for larger, controlled trials.

## Introduction

Chronic periodontitis is among the most prevalent inflammatory conditions worldwide and remains a major public health concern. While scaling and root planing (SRP) is well established as the first-line non-surgical therapy, its effectiveness can be limited by residual inflammation, microbial recolonization, and the initial depth of periodontal pockets [1,2]. These challenges may be further compounded in patients with systemic risk factors, such as smoking, diabetes, or poor compliance, where healing can be delayed and long-term stability is less predictable.

To address these challenges, topical adjuncts have emerged as valuable complements to mechanical debridement. Ideal adjunctive agents should not only provide antimicrobial activity but also promote soft tissue healing, reduce inflammation, and improve patient experience. Among these, PerioCream, a novel mucoadhesive formulation containing NitrAdine®, has been developed to be applied after SRP in the early healing phase. PerioCream is designed as a topical mucoadhesive cream to be applied directly to the gingival tissues, rather than as a rinse or mouthwash. NitrAdine® has demonstrated broad-spectrum antimicrobial and anti-biofilm activity with favorable tissue tolerance across dental applications such as denture hygiene and implant mucositis [2-5]. Preliminary laboratory and clinical studies suggest reductions in microbial load and modulation of gingival microbiota, with good tissue tolerance; however, direct comparative data with chlorhexidine or silver diamine fluoride remain limited and require further investigation [6-9]. Early clinical experience with a NitrAdine-based brushing solution also showed benefits in gingivitis management [10]. These properties support its potential use as a post-SRP adjunct aimed at stabilizing subgingival conditions and enhancing healing [5].

In addition to clinical outcomes, there is increasing recognition of the importance of patient-centered endpoints in periodontal therapy. Tools such as the Visual Analog Scale (VAS) and Health-Related Quality of Life (HRQOL) questionnaires provide valuable insight into the real-world impact of treatment on discomfort, function, and daily life [11,12]. Furthermore, artificial intelligence (AI)-based imaging tools may offer objective, standardized ways to document soft-tissue changes over time, enhancing clinical follow-up and remote monitoring [13]. Their integration could enhance the precision of outcome assessment and provide quantifiable visual data complementary to traditional indices. However, the practical use of such tools is still limited by the need for validation against clinical gold standards and sensitivity to variations in image acquisition.

This observational study explores the use of PerioCream as an adjunct to SRP in a real-world clinical setting. The primary aim was to assess clinical safety and short-term improvements in inflammation and discomfort. The secondary objective was to evaluate the feasibility of incorporating AI-based visual analysis of intraoral photographs as a complementary outcome measure.

## Materials and methods

Study design and setting

This single-center, retrospective, non-interventional observational analysis used fully anonymized data from routine clinical care in Italy. The analysis covered patients treated between March and June 2025. No randomization, blinding, or procedures beyond the standard of care were undertaken; the topical adjunct (PerioCream) was used as part of routine practice, not as a protocolized intervention.

Participants

Fifteen adult patients (8 females, 7 males; age range, 35-64 years) with moderate-to-severe periodontitis (Stage II/III, Grade B/C) were identified via consecutive chart review and included in the analysis. Inclusion criteria were generalized gingival inflammation, Bleeding on Probing (BOP ≥ 20%), and ≥20 natural teeth. Exclusion criteria included pregnancy, smoking, ongoing antibiotic or anti-inflammatory therapy, uncontrolled systemic conditions (e.g., diabetes, cardiovascular disease), or periodontal surgery within the previous six months.

Treatment protocol

After a full-mouth SRP, performed in a single session by experienced periodontists, patients applied PerioCream (a NitrAdine®-based mucoadhesive cream) once daily for seven consecutive days, preferably at night, directly to the gingival margins using a sterile applicator. Patients were instructed to maintain standard home oral hygiene, consisting of twice-daily toothbrushing and interdental cleaning. No other adjunctive professional or pharmacological therapies were permitted during the observation period.

Clinical outcome measures

Gingival status was assessed using the Gingival Index (GI) as described by Löe and Silness [14]. BOP was recorded as the percentage of sites bleeding within 10 seconds after gentle probing (~0.25 N), following the method of Ainamo and Bay [15]. Patient-reported discomfort was evaluated using a 0-10 VAS, where 0 indicates no pain and 10 indicates the worst possible pain [16]. HRQOL was assessed using a study-specific questionnaire developed for this project, structured on conceptual domains derived from validated periodontal HRQOL instruments such as the OHIP-14 [11,12]. The questionnaire included items related to oral function (chewing, speaking), oral symptoms (pain, gingival sensitivity), psychosocial aspects (social confidence, emotional well-being), and general well-being (sleep quality, comfort). Both patient- and clinician-reported outcomes were collected using these standardized domains. Questionnaires were developed for this study based on relevant literature, without reproducing copyrighted items from any published instrument. Full details of the instruments used and the permissions statement are provided in Appendix A.

AI-based image analysis

Standardized intraoral digital photographs of the anterior region (maxillary and mandibular) were taken at baseline (T0) and after 72 hours (T3) under consistent lighting, distance, and framing conditions, using the same digital camera and settings for all patients. Images were analyzed using a pre-trained convolutional neural network (CNN) model previously validated for mucosal surface assessment. The algorithm generated color-coded heatmaps highlighting regions of inflammation/redness and produced a quantitative metric (red pixel ratio) as an index of soft-tissue inflammation. These outputs were compared with clinical indices to explore alignment between subjective clinical assessments and objective AI-based measurements. While this approach provides an additional quantitative perspective, it remains exploratory and may be influenced by variations in image capture and the lack of a formal gold standard for validation in this specific dataset.

Statistical analysis 

Continuous variables were summarized as mean ± SD for normally distributed data or as median (IQR) for non-normal distributions. Absolute and relative changes (Δ, Δ%) were also calculated where appropriate. Paired comparisons between baseline (T0) and 72 hours (T3) for continuous and ordinal outcomes (BOP, GI, VAS) were performed using the two-tailed Wilcoxon signed-rank test. For each test, W and standardized Z values were reported, together with effect size (r = Z/√N). Categorical paired data were analyzed using the Stuart-Maxwell test for multi-level responses and McNemar’s test for binary outcomes, with χ², degrees of freedom (df), and exact p-values reported. HRQOL domains were collected only at T3; therefore, no paired HRQOL tests were conducted, and results were reported descriptively. Statistical significance was set at α = 0.05, with values of p < 0.001 reported explicitly. All analyses were performed using IBM SPSS Statistics for Windows, Version 25.0 (Released 2017; IBM Corp., Armonk, NY, USA).

## Results

All fifteen patients completed the 72-hour follow-up and adhered to the PerioCream application protocol. No adverse effects, allergic reactions, or treatment discontinuations were reported.

Clinical outcomes 

Periodontal parameters improved over 72 hours. BOP declined from 72% (70.5-74.0) at T0 to 12% (11.0-13.5) at T3 (Wilcoxon W = 0, Z = −3.41, p < 0.001; r = 0.88). GI decreased from 1.6 (1.55-1.7) to 0.5 (0.4-0.5) (W = 0, Z = −3.41, p < 0.001; r =0.88). Detailed distributions (mean ± SD and median (IQR)) (r = |Z|/√N; N = 15) are reported in Table 1 and Table 2.

**Table 1:** Summary of BOP and GI at baseline (T0) and 72 hours (T3) Values shown in cells are means; distributions are also summarized as median (IQR). Paired T0 vs T3 comparisons used the two-tailed Wilcoxon signed-rank test with W and standardized Z reported. BOP: Bleeding on Probing, GI: Gingival Index.

Parameter	T0 (baseline)	T3 (72 hours)	Absolute change	% change	p-value
BOP (%), mean	72.34	12.3	-60.04	-83.02%	<0.001
GI, mean	1.65	0.46	-1.19	-72.12%	<0.001

**Table 2:** Individual BOP and GI scores of the 15 patients Per-patient paired values for BOP (%) and GI (0-3) at baseline (T0) and 72 hours (T3); where space permits, absolute change is shown (Δ = T3 - T0). No hypothesis testing was performed at the individual level; group-level comparisons used the Wilcoxon signed-rank test and are reported in Table 1. BOP: Bleeding on Probing; GI: Gingival Index.

Patient ID	BOP (%) at T0	BOP (%) at T3	GI at T0	GI at T3
1.0	70.0	15.0	1.6	0.5
2.0	74.0	10.0	1.7	0.4
3.0	69.0	20.0	1.5	0.6
4.0	76.0	12.0	1.8	0.5
5.0	73.0	11.0	1.6	0.3
6.0	71.0	14.0	1.7	0.5
7.0	75.0	13.0	1.5	0.4
8.0	70.0	12.0	1.7	0.6
9.0	72.0	10.0	1.6	0.5
10.0	74.0	9.0	1.5	0.4
11.0	73.0	11.0	1.6	0.5
12.0	71.0	14.0	1.7	0.6
13.0	70.0	13.0	1.5	0.4
14.0	72.0	12.0	1.6	0.5
15.0	75.0	11.0	1.7	0.3

Patient-reported outcomes 

Patients reported early improvements in comfort within three days. The mean pain VAS decreased from 3.53 (T0) to 2.27 (T3), not reaching statistical significance (Wilcoxon W = 21.0, Z = 1.42, p = 0.157), and HRQOL responses shifted toward more favorable categories across pain, eating/speaking comfort, sleep quality, and social confidence, as summarized in Table 3.

**Table 3:** Patient‑reported outcomes (HRQOL domains and VAS) HRQL domains are reported as n and % of N = 15 per category at T3. Pre-/post-HRQOL categories were not collected; therefore, no hypothesis testing was performed for HRQOL items. The VAS (0-10) pain/discomfort item was recorded at T0 and T3 and is summarized as mean ± SD and median (IQR). The two-tailed Wilcoxon signed-rank test was used for T0 vs T3 with W, standardized Z, and p reported. Statistical significance α = 0.05 (values p < 0.001 reported explicitly).

Section	Domain/measure	Category/metric	T0 value	T3 value	Test statistic	df	p-value
HRQOL	Eating difficulty	No difficulty	-	13 (86.7%)	-	-	-
HRQOL	Eating difficulty	Slight difficulty	-	1 (6.7%)	-	-	-
HRQOL	Eating difficulty	Moderate difficulty	-	1 (6.7%)	-	-	-
HRQOL	Eating difficulty	Severe difficulty	-	0 (0.0%)	-	-	-
HRQOL	Speaking comfort	Very comfortable	-	3 (20.0%)	-	-	-
HRQOL	Speaking comfort	Slightly uncomfortable	-	8 (53.3%)	-	-	-
HRQOL	Speaking comfort	Moderately uncomfortable	-	2 (13.3%)	-	-	-
HRQOL	Speaking comfort	Very uncomfortable	-	1 (6.7%)	-	-	-
HRQOL	Gingival sensitivity	Yes, completely	-	11 (73.3%)	-	-	-
HRQOL	Gingival sensitivity	Yes, partially	-	3 (20.0%)	-	-	-
HRQOL	Gingival sensitivity	No change	-	1 (6.7%)	-	-	-
HRQOL	Adverse events	No irritation	-	14 (93.3%)	-	-	-
HRQOL	Adverse events	Mild irritation	-	0 (0.0%)	-	-	-
HRQOL	Adverse events	Moderate irritation	-	0 (0.0%)	-	-	-
HRQOL	Adverse events	Severe irritation	-	0 (0.0%)	-	-	-
HRQOL	Recommendation	Yes, absolutely	-	13 (86.7%)	-	-	-
HRQOL	Recommendation	Maybe	-	1 (6.7%)	-	-	-
HRQOL	Recommendation	No	-	1 (6.7%)	-	-	-
VAS	Pain/discomfort (0-10)	Mean ± SD; median (IQR)	3.53 ± 3.29; 3 (0-6)	2.27 ± 3.24; 1 (0-3)	W = 21.0; Z = 1.42	-	0.157

Clinician feedback and compliance 

Clinicians reported high usability and muco-adhesion: application was rated easy or very easy in 14/15 (93.3%) cases (8/15 (53.3%) easy; 6/15 (40.0%) very easy), with 1/15 (6.7%) moderate. Gingival adhesion was very good in 13/15 (86.7%) and fair in 2/15 (13.3%), and a visible protective barrier formed in 15/15 (100%). Bleeding reduction was judged significant in 12/15 (80.0%) and slight in 3/15 (20.0%). In clinician-perceived patient feedback, pain compared with prior SRP was lower in 12/15 (80.0%) (5/15 (33.3%) much lower; 7/15 (46.7%) slightly lower) and unchanged in 3/15 (20.0%); speaking/eating comfort improved in 5/15 (33.3%) (2/15 (13.3%) significant; 3/15 (20.0%) slight), showed no change in 9/15 (60.0%), and worsened in 1/15 (6.7%). Adverse events were absent in 14/15 (93.3%), with one moderate event (6.7%) (Table 4).

**Table 4:** Clinician observations and patient-reported feedback (perceived by clinicians) Post-treatment ratings are reported as n and % (N = 15) for each item/response level (e.g., ease of application, gingival adhesion, bleeding reduction). Percentages are computed on non-missing responses and, for single-select items, totals ≈100% (minor deviations due to rounding). No formal hypothesis testing was performed (descriptive endpoints). When shown, 95% confidence intervals (CIs) for proportions are provided in parentheses (Wilson method).

Domain	Item/aspect	Count (n)	% (N = 15)
Clinician observations	Ease of application: easy	8	53.3%
	Ease of application: very easy	6	40.0%
	Ease of application: moderate	1	6.7%
	Gingival adhesion: very good	13	86.7%
	Gingival adhesion: fair	2	13.3%
	Bleeding reduction: significant	12	80.0%
	Bleeding reduction: slight	3	20.0%
	Visible barrier formed	15	100%
Patient-reported feedback (as perceived by clinicians)	Pain vs prior SRP: much lower	5	33.3%
	Pain vs prior SRP: slightly lower	7	46.7%
	Pain vs prior SRP: no difference	3	20.0%
	Speaking/eating comfort: no change	9	60.0%
	Speaking/eating comfort: slight improvement	3	20.0%
	Speaking/eating comfort: significant improvement	2	13.3%
	Speaking/eating comfort: worsening	1	6.7%
	Reported adverse events: none	14	93.3%
	Reported adverse events: moderate	1	6.7%

AI-based image analysis 

AI-assisted image analysis supported the clinical findings: 13/15 patients showed a visible reduction in redness and edema from T0 to T3 (72 hours), and CNN heatmaps demonstrated a decreased inflammatory signal in the anterior gingiva. These results indicate that objective, image-based methods can complement traditional indices (BOP, GI) for short-term monitoring of gingival healing [13].

## Discussion

This observational study investigated the short-term effects of PerioCream as an adjunctive treatment following SRP, with a 72-hour follow-up period. The results support the hypothesis that topical NitrAdine®-based therapy may enhance early gingival healing and improve patient-reported outcomes when applied immediately after mechanical debridement.

Within just three days, significant reductions were observed in both BOP and GI, indicating an early resolution of inflammation. The significant reduction in inflammation observed within 72 hours may suggest a short-term substantivity of the mucoadhesive NitrAdine® formulation, likely related to its ability to prolong local bioavailability at the gingival margin. However, this observation remains preliminary and requires confirmation in longer-term studies with pharmacokinetic and microbiological evaluation. These outcomes align with the known antimicrobial and anti-inflammatory properties of NitrAdine®, previously documented in various clinical settings, including denture-related stomatitis and peri-implant mucositis [3,4,7]. Its ability to disrupt biofilm, reduce microbial load, and avoid common side effects of chlorhexidine, such as taste alteration and staining, makes it a valuable candidate for non-surgical periodontal protocols [2,6,9].

Importantly, improvements were not limited to clinical indices. Patient-reported data indicated reduced discomfort and better daily function, particularly for eating, speaking, and social confidence, consistent with the growing emphasis on patient-centered outcomes in periodontal care [11,12].

The use of AI-assisted image analysis in this study provided supportive evidence in line with the clinical findings. The CNN-based model detected reductions in mucosal redness consistent with improvements in GI. Such tools may help standardize the documentation of inflammation and could support asynchronous or remote monitoring, particularly in preventive and maintenance phases of periodontal therapy [13]. However, while AI analysis should not replace clinical judgment, it may complement it by offering objective, quantifiable visual data. These findings should be regarded as exploratory and require validation in larger and more diverse datasets.

Despite encouraging findings, this study has important limitations: the small sample (n = 15), the absence of an SRP-only control, and the brief 72-hour follow-up, which precludes conclusions about durability. Although multicenter, all clinics were within a single country, potentially limiting generalizability. Nonetheless, the consistent early improvements in BOP, GI, and patient-reported outcomes, together with AI-assisted image analysis as an objective adjunct, support the feasibility and tolerability of PerioCream in the immediate post-SRP phase and justify larger, controlled trials with extended follow-up and external validation of image-based endpoints.

## Conclusions

This real-world observational study suggests that the adjunctive use of PerioCream, a NitrAdine®-based topical formulation, may offer potential short-term benefits in the early post-treatment phase following SRP. Within 72 hours, reductions in gingival inflammation were observed, both objectively, through decreases in BOP and GI, and subjectively, through improvements in patient comfort and self-reported well-being. While these early results are encouraging, they remain preliminary due to the small sample size, absence of a control group, and short follow-up and should be interpreted with caution until confirmed by larger, controlled studies.

The product was well tolerated, easy to apply, and positively received by both patients and clinicians. The integration of AI-assisted image analysis offered additional support to the clinical findings, highlighting the potential value of digital tools for documentation and follow-up. Nonetheless, the study is limited by its small sample size, lack of a control group, and short follow-up period. These findings should therefore be regarded as preliminary. Future research should include larger, controlled clinical trials with extended observation periods and validation of digital outcome measures. The incorporation of patient-centered endpoints and AI-based evaluations represents a forward-looking approach to periodontal care that may enhance precision, personalization, and long-term adherence.

## Appendices

Appendix A

Instruments and Permissions Statement

The Gingival Index [14], Bleeding on Probing [15], and Visual Analog Scale for pain [16] are standard clinical or patient-reported measures; the original sources are cited in the Methods section.

The HRQOL, patient, and clinician questionnaires were designed specifically for this study, based on conceptual domains from OHIP-14 and related periodontal HRQoL literature [11,12]. Wording and structure were original to this study. No copyrighted questionnaire items were reproduced; therefore, no permission was required.

**Table 5:** Patient questionnaire (post‑SRP with PerioCream) Self‑developed instrument used in this observational study; VAS recorded at T0 and T3; HRQOL items at T3 only. SRP: scaling and root planing, VAS: Visual Analog Scale.

Section/item	Question	Response options
Section 1: pain and discomfort	1. On a scale from 0 to 10, how much pain or discomfort did you experience immediately after the SRP procedure?	0, no pain; 10, worst pain
	2. Compared to previous experiences with dental cleanings or gum treatments, how would you rate your discomfort today?	Much less discomfort/Slightly less/About the same/More than usual
	3. Did you need to take anything (e.g., a pain reliever or home remedy) after the procedure to relieve discomfort?	Yes/No
Section 2: functional impact	4. Did you experience any difficulty eating after the SRP treatment?	None/Mild/Moderate/Severe
	5. How comfortable did you feel when speaking after the treatment?	Very comfortable/Slightly uncomfortable/Moderately uncomfortable/Very uncomfortable
	6. Did you experience gum sensitivity or discomfort in the hours following the treatment?	None/Mild/Moderate/Severe
Section 3: well-being and satisfaction	7. On a scale from 0 to 10, how satisfied are you with your overall experience of the SRP treatment?	0, not at all satisfied; 10, extremely satisfied
	8. Do you believe a protective or soothing gum product applied after SRP could improve post-treatment comfort?	Yes, definitely/Maybe/No
	9. Additional comments (optional)	Free text

**Table 6:** Clinician questionnaire (post-SRP with PerioCream) Self-developed instrument used in this observational study; completed by treating clinicians at T3. SRP: scaling and root planing.

Section/item	Question	Response options
1. Clinical observations	1. How much bleeding was observed immediately after SRP?	None/Mild/Moderate/Heavy
	2. Clinical response of the gingival tissue post-SRP	Good/Moderate/Poor
2. Patient perception (clinically observed)	3. Did the patient show signs of pain or discomfort after the treatment?	No/Mild/Moderate/Severe
	4. Did the patient ask for something to relieve post-SRP discomfort?	No/Yes
3. Clinician’s opinion	5. Do you think a soothing product after SRP would be useful?	Yes, absolutely/Maybe/No
	6. Comments or clinical notes	Free text
